# Rapid detection of *Puccinia triticina* causing leaf rust of wheat by PCR and loop mediated isothermal amplification

**DOI:** 10.1371/journal.pone.0196409

**Published:** 2018-04-26

**Authors:** C. Manjunatha, Sapna Sharma, Deepika Kulshreshtha, Sangeeta Gupta, Kartar Singh, Subhash C. Bhardwaj, Rashmi Aggarwal

**Affiliations:** 1 Fungal Molecular Biology Laboratory, Division of Plant Pathology, ICAR—Indian Agricultural Research Institute, New Delhi, India; 2 ICAR-National Bureau of Plant Genetic Resources, New Delhi, India; 3 ICAR—Indian Institute of Wheat and Barley Research, Regional Station, Flowerdale, Shimla, India; Institute of Genetics and Developmental Biology Chinese Academy of Sciences, CHINA

## Abstract

Leaf rust of wheat caused by *Puccinia triticina* has significant impact on wheat production worldwide. Effective and quick detection methodologies are required to mitigate yield loss and time constraints associated with monitoring and management of leaf rust of wheat. In the present study, detection of *P*. *triticina* has been simplified by developing a rapid, reliable, efficient and visual colorimetric method *i*.*e*., loop mediated isothermal amplification of DNA (LAMP). Based on *in silico* analysis of *P*. *triticina* genome, PTS68, a simple sequence repeat was found highly specific to leaf rust fungus. A marker (PtRA_68_) was developed and its specificity was validated through PCR technique which gave a unique and sharp band of 919 bp in *P*. *triticina* pathotypes only. A novel gene amplification method LAMP which enables visual detection of pathogen by naked eye was developed for leaf rust pathogen. A set of six primers was designed from specific region of *P*. *triticina* and conditions were optimised to complete the observation process in 60 minutes at 65^o^ C. The assay developed in the study could detect presence of *P*. *triticina* on wheat at 24 hpi (pre-symptomatic stage) which was much earlier than PCR without requiring thermal cycler. Sensitivity of LAMP assay developed in the study was 100 fg which was more sensitive than conventional PCR (50 pg) and equivalent to qPCR (100 fg). The protocol developed in the study was utilized for detection of leaf rust infected samples collected from different wheat fields. LAMP based colorimetric detection assay showed sky blue color in positive reaction and violet color in negative reaction after addition of 120 μM hydroxyl napthol blue (HNB) solution to reaction mixture. Similarly, 0.6 mg Ethidium bromide/ml was added to LAMP products, placed on transilluminator to witness full brightness in positive reaction and no such brightness could be seen in negative reaction mixture. Further, LAMP products spread in a ladder like banding pattern in gel electrophoresis. Our assay is significantly faster than the conventional methods used in the identification of *P*. *triticina*. The assay developed in the study shall be very much useful in the development of diagnostic kit for monitoring disease, creation of prediction model and efficient management of disease.

## 1. Introduction

Wheat (*Triticum aestivum* L.) is an important cereal crop worldwide and is grown in an area of about 222.28 million hectares in a range of environments with 724 million tonnes production (FAO, 2016). Leaf rust of wheat caused by *Puccinia triticina* is considered as one of the major constraints affecting wheat production across the world [1]. For effective management of the leaf rust of wheat timely detection of pathogen is need of the hour. Traditional disease diagnostic methods require elaborate screening under artificial epiphytotic conditions which is time consuming and involves tedious protocols. These traditional methods can detect pathogen after the appearance of the symptoms. *P*. *triticina* takes about 7 to 10 days for symptom development during infection. During this time, symptoms may merge with stripe rust pustules; hence it becomes very difficult to differentiate two types of rusts under field conditions. Further, specific molecular marker can be utilized for accurate forecasting of early disease outbreak by determining the initial inoculum potential of the pathogen, so that prophylactic disease management strategy can be taken to reduce losses caused by pathogen [2]. Thus, there is a need for development of specific, sensitive and reliable molecular marker for early detection of pathogen which may assist in better management of the disease. PCR based diagnostic assays have been developed for detection of *Puccinia striiformis* f. sp. *tritici* causing stripe rust of wheat [2, 3], *Bipolaris sorokiniana* causing spot blotch of wheat [4], *Fusarium graminearum* complex in wheat [5], *Puccinia graminis* f.sp. *tritici* [6]. However PCR based detection for leaf rust pathogen is not available till date. Thus, there is a need for development of specific and sensitive molecular marker for early detection of the pathogen which may assist in better management of the disease. Although, PCR is the most commonly used DNA amplification method for detection of plant pathogens, however, expensive thermo cycler is required for amplification of DNA [7]. Recent advances in *in vivo* DNA synthesis confirmed the possibility of amplification of DNA under isothermal conditions without expensive thermal cycler using loop mediated isothermal amplification (LAMP) [8]. LAMP is a novel, innovative, unique gene amplification method emerging as a simple, rapid and highly sensitive diagnostic tool for early detection of plant pathogens [8, 9]. Amplification involves four sets of specially designed primers which identify six distinctive sequences of a gene of interest [10]. Primerssize and sequences are chosen in such a manner that *Tm* of primers fall between 60^°^C and 65^°^C [11] so that amplification of target gene can be completed in a simple water bath under isothermal conditions [12]. DNA polymerase used in this method has strand displacement activity [13]. Amplified products can be detected by agarose gel electrophoresis or real time monitoring in an inexpensive turbidometer or simply by naked eye as turbidity changes upon amplification [9]. Presence of target pathogen can be indicated by presence of amplified product as it is highly specific [11]. Both amplification and detection of gene of interest can be completed in a single step and in a single tube [14]. Because of fast amplification and simple procedure, LAMP has potential applications in detection of plant pathogens and screening of germplasm for disease without requiring thermal cycler. LAMP based diagnostic protocols have been developed for specific detection of *Erwinia amylovora* [15], *Ralstonia solanacearum* [16], *Napier stunt phytoplasma* in Napier grass [17], Potato virus Y [18], but very limited examples are available for fungal plant pathogens. Hence the aim of this study was to develop PCR and LAMP based diagnostic assay for effective and quick detection of *Puccinia triticina* infection in wheat which distinguishes leaf rust from other fungal diseases.

## 2. Materials and methods

### 2.1 Multiplication of rusts and other fungal pathogens of wheat

Ten pathotypes of leaf rust (12, 12–1, 12–2, 77, 77–1, 77–2, 77–5, 77–6, 77–8, 104–2), three pathotypes of stripe rust (46S119, 38S102 and 78S84) and two pathotypes of stem rust (40–1 and 40-A) of wheat were collected from ICAR- Indian Institute of Wheat and Barley Research (IIWBR) Regional Station Flowerdale, Shimla, India. The single uredial culture of each rust pathotype was multiplied on Agra local, a susceptible cultivar under glass house conditions. The seedlings at one leaf stage were inoculated with *P*. *triticina* pathotype individually. After inoculation the plants were kept in humid chambers with diffused light for 48 hours then the plants were shifted to glass house and maintained upto symptom development. All through the experiment the temperature of glass house was maintained at 22^°^C. The spore mass was collected and stored at -20^°^C [2]. Likewise, *Blumeria graminis tritici* was also maintained on the susceptible host (Agra local). Other pathogens like *Bipolaris sorokiniana* (BS-75), *Bipolaris oryzae* (BO-1) and *Fusarium graminearum* were cultured on potato dextrose broth (PDB) at 25^°^C for one week at 120 rpm. Fungal mycelium was filter sterilized and stored at -20°C for further use (Table 1).

**Table 1:** Details of pathotypes used in the study.

Sl No	Name of pathogen	Name of pathotype	Place of origin	Year of report
1	*Puccinia triticina*	(12)	Thordi	1966
2	*Puccinia triticina*	(12–1)	Gwalior	1983
3	*Puccinia triticina*	(12–2)	Hansi	1979
4	*Puccinia triticina*	(77–4)	Nilgiris	1989
5	*Puccinia triticina*	(77–1)	Nilgiris	1985
6	*Puccinia triticina*	(77–2)	Nilgiris	1984
7	*Puccinia triticina*	(77–5)	Nilgiris	1992
8	*Puccinia triticina*	(77–6)	Nilgiris	1997
9	*Puccinia triticina*	(77–8)	Arabhavi	2004
10	*Puccinia triticina*	(104–2)	Malan	1991
11	*Puccinia striiformis* f. sp. *Tritici*	(46S119)	Gurdaspur	1996
12	*Puccinia striiformis* f. sp. *Tritici*	(38S102)	Nilgiri hills	1973
13	*Puccinia striiformis* f. sp. *Tritici*	(78S84)	Batala (Gurdaspur) Punjab	2001
14	*Puccinia graminis* f. sp. *Tritici*	(40–1)	Wellington	1989
15	*Puccinia graminis* f. sp. *Tritici*	(40-A)	Wellington	1974
16	*Bipolaris sorokiniana*	BS-75		
17	*Bipolaris oryzae*	BO-1		
18	*Fusarium graminearum*	ITCC 3437		
19	*Blumeria graminis tritici*	-	Wellington	-

### 2.2 Extraction of genomic DNA

Genomic DNA was extracted from *P*. *triticina*, *P*. *striiformis* f. sp. *tritici* and *P*. *graminis* f. sp. *tritici* pathotypes using ZR soil microbe DNA miniprepkit (Zymo research, USA) following the manufacture’s protocol [2]. DNA from wheat leaf samples and other fungal pathogens was isolated by CTAB method [19]. One microliter of ribonuclease (10 mg/mL) was added to the extracted nucleic acid and stored at 4°C overnight to completely digest the RNA. DNA was quantified with an ND1000 Nanodrop spectrophotometer (Thermo Fisher Scientific, USA), and DNA concentration was adjusted to 50 ng/μL for PCR amplification. The DNA extracted was stored at -20^0^ C for further use.

### 2.3 *In silico* analysis of *Puccinia triticina* genome and designing of specific primers and their validation

Exploration of *P*. *triticina* genome sequences and available literature led to selection of some of the simple sequence repeat markers utilized [20] for diversity study of leaf rust pathogen. The uniqueness of the selected sequence was validated and confirmed by BLAST analysis showing no similarity with other *Puccinia* species or wheat pathogens. Primers were designed for selected sequence using *Primer3 plus* software and their quality was tested using IDT oligo analyser for the potential of secondary structure formation, self-complementation and dimer formation within and between different primers (Table 2).

**Table 2:** Details of the LAMP primers used in the development of diagnostic assay.

Name	Sequence	Length (bp)
**A**	**Conventional PCR Primers**	
PtRA_68_F	CTACTCAGCCAGAAGCACCTC	21
PtRA_68_R	TACTGACCACCAGCGTCTTG	20
**B**	**Real time PCR Primers**	
PtRA_68_(RT)F	CTGAGTCTGCTTGTAGTTGGG	21
PtRA_68_(RT)R	CTTGCTTTTCGGGCTTGATG	20
**C**	**LAMP Primers**	
F3	TCGACAGACTGAGTCTGCT	19
B3	CGATCTTGATGCGCATCTCC	20
FIP	TGTGCTGGATGGCGGATAGGAT-GTTGGGGTCATTCTCAACCG	42
BIP	CATCGGGACATCAAGCCCGAA-TCAACACAAAGTCAGCCGT	40
LF	GGCATAGAACCTGGCACAGT	20
LB	AAGCAAGCCTCCATTCTCACA	21

#### 2.3.1 Optimization of PCR conditions

Purified genomic DNA of *P*. *triticina* (pathotype 77–5) was utilized as template for optimization of PCR amplification. The PCR reaction was carried in 25 μl volume containing 25 to 75 ng of genomic DNA, 100 to 400 μM each dNTP (10mM), 1–10 μM primer, 1.5–2.5 mM 25 mM MgCl_2_ (Thermo Fisher Scientific), 1.5 U concentration of *Taq* DNA polymerase (Banglore Genei, India) and 1X *Taq* buffer. Amplification was performed in a thermal cycler (BioRAD, USA). Thermal cycler was programmed for one cycle of denaturation at 94^°^C for 5 min followed by 30 to 35 cycles of denaturation at 94°C for 1 min, annealing at 58 to 66^°^C for 1 min and extension at 72^°^C for 2 min. A final extension step at 72^°^C for 7 to 10 min was also performed. Sterilized distilled water was used as a negative control to test for the presence of contamination in PCR reagents.

#### 2.3.2 Cloning and sequencing of unique amplicon

*P*. *triticina* specific primers were selected based on PCR amplification and fragments were separated on 1.2% (w/v) agarose gel. A specific band of 919 bp was purified from the gel using the gel extraction kit (Rosche Life Sciences, United States) and this amplicon was cloned into the pGEMT easy vector (Promega Corporation, USA). Plasmids were sequenced and the sequence was BLAST analysed using NCBI database to check for its specificity.

#### 2.3.3 Specificity and sensitivity test of the marker

The specificity of marker was tested by performing PCR with genomic DNA of ten selected pathotypes of *P*. *triticina* mentioned earlier (Table 1) and one pathotype each of *P*. *graminis* f. sp. *tritici*, *P*. *striiformis* f. sp. *tritici*, *B*. *oryzae*, *Blumeria graminis tritici* and *B*. *sorokiniana*. PCR products were electrophoresed in 1.2% (w/v) agarose gel containing ethidium bromide (0.5μg/ml) in 1X TAE buffer. To test the sensitivity of marker, different concentrations of genomic DNA *viz*. 100ng, 50ng, 10ng, 1ng, 500pg, 100pg, 50pg and 25pg of *P*. *triticina* (pathotype 77–5) was used as DNA template for PCR amplification.

#### 2.3.4 Validation of the marker

For testing the performance of the marker leaf rust susceptible wheat cultivar (Agra local) was inoculated with *P*. *triticina* (pathotype 77–5) as per the procedure mentioned above. For mock inoculation plants were sprayed with water. Leaf samples were collected at 0, 24 h, 48 h, 72 h, 4d, 5 d and at symptomatic stage after inoculation and stored at -40°C. The efficiency of specific primers was further confirmed by taking symptomatic and symptomless wheat leaf samples from ICAR-Indian Agricultural Research Institute research farm. DNA was extracted from all the collected samples by CTAB method and the PCR amplification was performed with primer set PtRA_68_F/R using standardized protocol mentioned earlier.

### 2.4 Real-time PCR assay for detection of *Puccinia triticina*

#### 2.4.1 Design of qPCR primers

To design specific primers for qPCR assays to detect *P*. *triticina*, the sequence of specific marker (PtRA_68_) developed using conventional PCR was exploited. A primer set [PtRA_68_(RT)F/PtRA_68_(RT)R] was designed using IDT analyser software **(https://www.idtdna.com)**. An *in silico* test for primer specificity was done by running the primer sequences against the non-redundant GenBank database (Table 2).

#### 2.4.2 Optimization of qPCR conditions

Real time PCR conditions like annealing temperature, primer concentration and temperature to measure the fluorescence signal of specific amplicon using *P*. *triticina* DNA as template were optimized. The optimised qPCR assay consisted of 2μL (100-10fg) of template DNA, 1μl of the forward and reverse primers each (1μM), 10μl of PCR master mix with SYBR green buffer (Genetix, India) in a reaction volume of 20μl. The volume was adjusted with nuclease free water. All experiments were performed in triplicate.

Light Cycler conditions were as follows: 95°C for 5 min, 40 cycles of PCR amplification at 94°C for 15s and 50°C-55°C for 30s followed by default melt curve analysis. Double distilled water was used as a negative control.

#### 2.4.3 Development of standard curve

Standard curve for qPCR assay was developed from 10 fold dilution of *P*. *triticina* (pathotype 77–5) DNA (100ng/μl) by serial dilution in PCR grade water. The standard curve was generated by plotting the Ct values versus the logarithm of the quantity of the serially diluted genomic DNA. Standard regression lines were generated for each of DNA standard curves using a range of DNA from 100ng to 10fg. The standard regression line was used as a reference curve for transforming the experimental Ct values into amount of *P*. *triticina* DNA (fg). The amplification efficiency [E = 10 ^(-1/slope)^ -1] was calculated for the standard curve.

### 2.5 LAMP assay for detection of *Puccinia triticina*

#### 2.5.1 Designing of primers for LAMP detection assay

**Fig 1:**
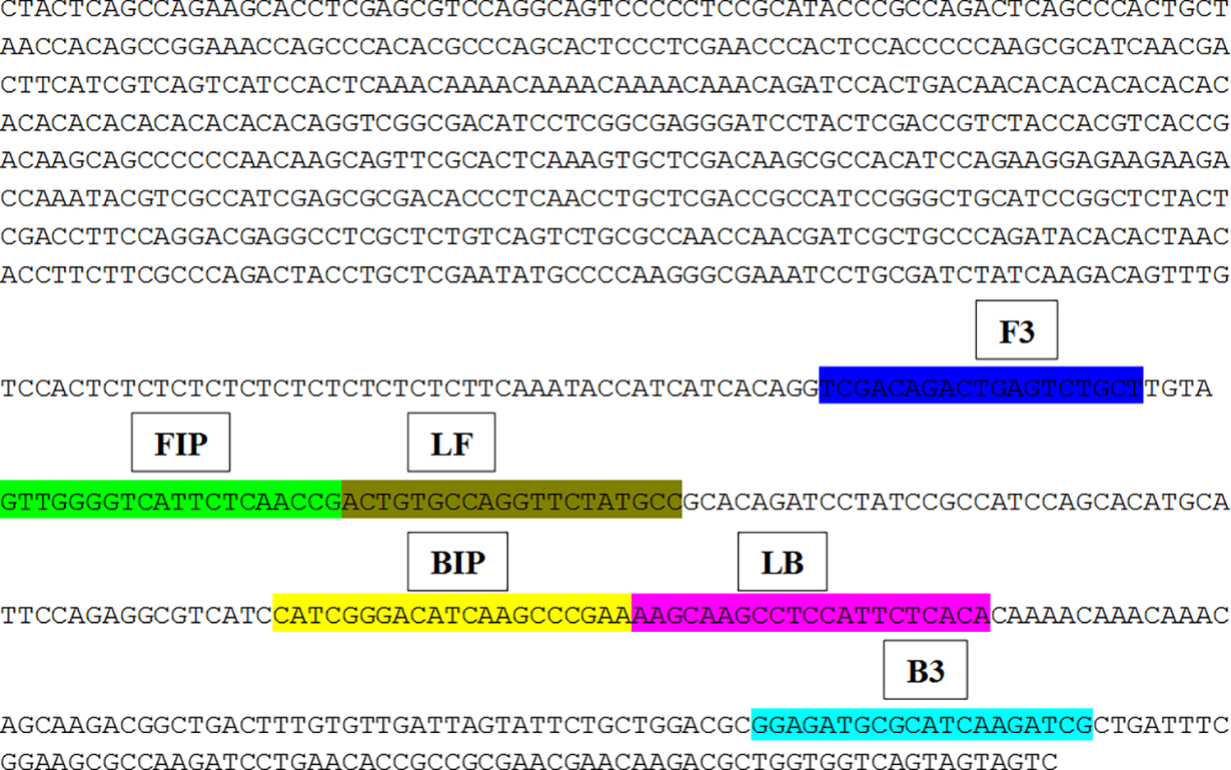
The structure of the LAMP primers and their complementarity to target DNA.

Six primers were designed based on specific sequence (PtRA_68_) for early detection *P*. *triticina* using *Primer Explorer V4* software (http://primerexplorer.jp/e). LAMP primers used in the study are listed in Table 2. The structure of the LAMP primers and their complementarity to target DNA used in the study are shown in Fig 1.

#### 2.5.2 Standardization of LAMP detection assay

The LAMP detection assay was performed in a 25 μl reaction volume. For standardization of protocol, concentrations of 25mM MgSO_4_ (2–4 μl) and 10mM dNTPs (3–4 μl) were taken. Concentrations of inner and outer primers to be used were also optimized. Betaine 5μl (Sigma) and 8U *Bst polymerase* (New England Biologicals, USA) along with buffer was also added in the reaction mixture. The reaction mixture was pre-heated to 95^°^C for 5 minutes and incubated at 65°C for 30 to 120 min using a dry bath (Banglore Genie, India); further for termination of the reaction, the mixture was heated at 80°C for 10 min.

#### 2.5.3 Colorimetric detection of *Puccinia triticina*

The change in turbidity of LAMP amplified products were visually observed. Subsequently, 100 to 200 μM HNB dye was added to 10 μl LAMP amplified products, to test the change in color reaction mixture. Further, 0.1 to 0.8 mg ethidium bromide/ml (Sigma), was added to 10 μl amplified products to observe difference in brightness under a UV transilluminator. Finally banding pattern of LAMP products were noted by resolving on 2% (w/v) agarose gel.

#### 2.5.4 Specificity and sensitivity test of LAMP assay

For determining specific amplification of *P*. *triticina* in LAMP assay, same primer sets were used to amplify the genome of *P*. *striiformis* f. sp. *tritici*, *P*. *graminis* f. sp *tritici*, *B*. *sorokiniana*, *B*. *oryzae*, *Blumeria graminis tritici*, *F*. *graminearum* and host genome (healthy wheat leaves). To test the sensitivity of the LAMP assay total genomic DNA of *P*. *triticina* diluted to different concentrations *viz*. 100ng, 50ng, 10ng, 1ng, 100pg, 50pg, 10pg, 100fg, 10fg and nuclease free water as non-template control (NTC) were taken. LAMP assay was carried out at optimized conditions.

#### 2.5.5 Validation and utilization of of LAMP assay for detection of *Puccinia triticina*

Samples were taken from *P*. *triticina* (77–5) inoculated wheat leaves at different time points (0hr, 24hr and 48hr). LAMP assay was performed by taking nuclease free water as non-template control (NTC) and genomic DNA of *P*. *triticina* as positive control. The effectiveness of specific primers was further confirmed using field samples. DNA extracted was subjected to LAMP assay. The assay was performed at conditions optimized earlier.

## 3. Results

### 3.1 PCR amplification, cloning and sequencing of the marker

*In silico* analysis of *P*. *triticina* genome and exploration of simple sequence repeats available in the database showed accession number DQ789152 to be highly specific to *P*. *triticina* in NCBI database after BLAST analysis. Primers designed from accession DQ789152 produced a unique band of 919 bp in *P*. *triticina* pathotypes only. The standardised PCR protocol consisted of 25 ng of genomic DNA, 400μM each dNTP, 10 μM primer, 2.5 mM MgCl_2_, 1.5 U*Taq* DNA polymerase and 1X*Taq* buffer. The optimum annealing temperature was 60°C. The unique amplicon of 919 bp cloned into pGEMT easy vector was sequenced. The sequencing resuts showed primer sequence at both the ends. No ORF was found in this fragment. After subsequent elimination of vector sequence, a sequence of 919bp specific to *P*. *triticina* was obtained. The sequence (PtRA_68_) was submitted in GenBank with accession number KY747393.

#### 3.1.1 Specificity and sensitivity test of marker

**Fig 2:**
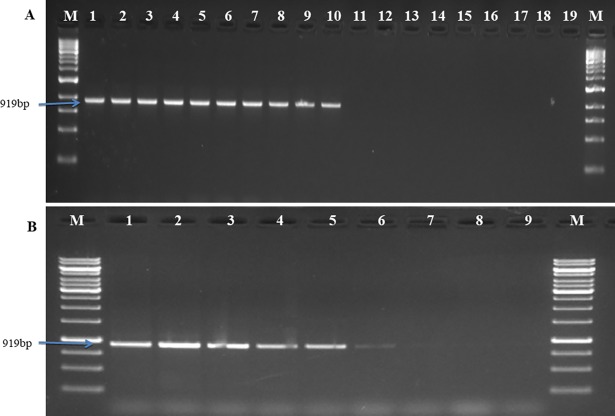
**Agarose gel showing specificity (A) and sensitivity (B) using PtRA**_**68**_**F/R primers set.** (A) Specificity test using DNA templates of different *Puccinia* spp. Lanes: M: 1 kb ladder, Fermentas; 1–10: DNA template of *P*. *triticina* (1–12, 2-12-1, 3-12-2, 4–77, 5-77-1, 6-77-2,7-77-5, 8-77-6, 9-77-8, 10-104-2); 11–13: *P*. *striiformis* f. sp. *tritici* (46S119, 38S102 and 78S84); 14–15: *P*. *graminis* f. sp. *tritici* (40–1 and 40-A); 16-*Bipolaris sorokiniana* (BS-75); 17: *B*. *oryzae* (BO-1); 18:-Healthy wheat leaf DNA; 19: sterile water. (B) Sensitivity test using different concentration of template DNA. Lanes: M: 1kb ladder, Fermentas; 1: 100ng; 2: 50ng; 3: 10ng; 4: 1ng; 5: 500pg; 6: 100pg; 7: 50pg; 8: 25pg DNA as a template, 9: sterile water.

The specificity of the marker developed in the study was examined by standardized PCR protocol with genomic DNA of *P*. *triticina* and other species of *Puccinia* as well as other wheat pathogens. Primer set [PtRA_68_ F/R produced highly specific band of 919 bp in *P*. *triticina*, differenting this fungus from other fungal pathogens (Fig 2A). The sensitivity of the marker (PtRA_68_) was 50 pg of *P*. *triticina* DNA. (Fig 2B).

#### 3.1.2 PCR based detection of *P*. *triticina* at different time intervals of inoculation and from field samples

**Fig 3:**
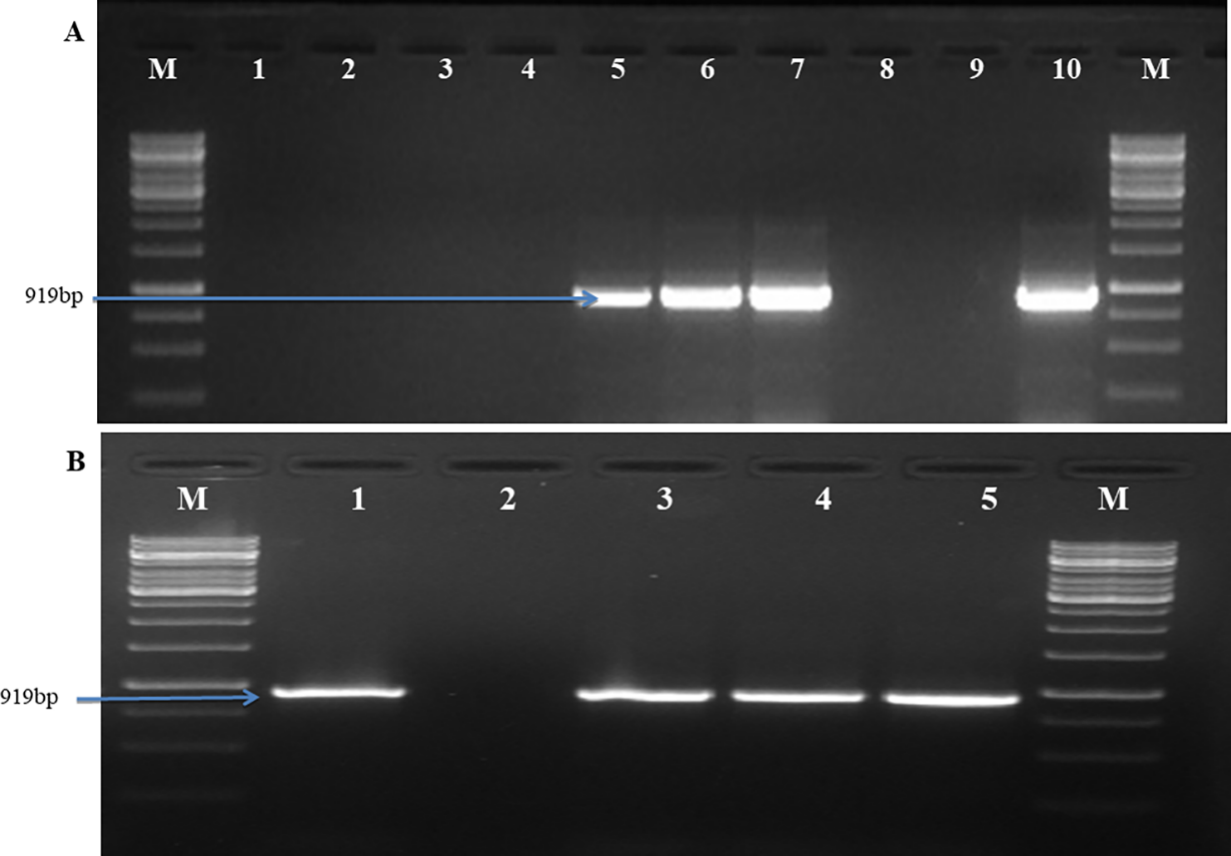
**PCR based detection of *P*. *triticina* using primer pair PtRA**_**68**_**F/R in (A) Glass house conditions (B) Field samples.** (A) PCR detection of *P*. *triticina* in leaves of wheat seedlings of ‘Agralocal’ at different time points after inoculation. Lanes: M—1 kb ladder (Fermentas); 1–6: genomic DNA of wheat after 0, 1, 2, 3, 4, 5 days after inoculation; 7: symptomatic leaf; 8: healthy wheat leaf DNA; 9: sterile water; 10: *P*. *triticina* DNA (positive control). (B) PCR detection of *P*. *triticina* in different field samples of ICAR-IARI, New Delhi. Lanes: M (1kb molecular marker, Fermentas); 1–4: PCR amplified product of different samples. 1, 3, 4 showing the presence of *P*. *triticina*; 2: absence of *P*. *triticina*; 5:genomic DNA of *P*. *triticina* (positive control).

PCR based detection assay diagnosed leaf rust of wheat at 4 days post inoculation by producing specific band of 919bp while no band was observed from uninoculated plants and negative control (NTC) (Fig 3A). Among 4 wheat leaf samples collected from field of ICAR-IARI New Delhi, 3 samples showed presence of *P*. *triticina* by amplifying a band of 919bp. So the marker developed in the study validated its effectiveness to diagnose leaf rust of wheat directly from wheat field (Fig 3B).

### 3.2 Real time PCR based detection

**Fig 4:**
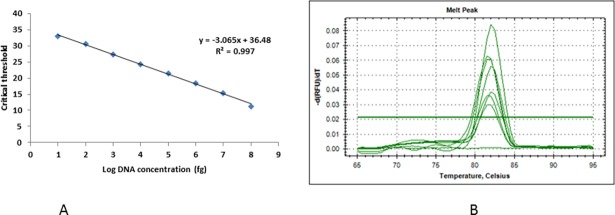
Real time PCR analysis. (A) Standard curve showing the log_10_ DNA amount (fg) plotted against cycle threshold (Ct) for different dilutions of genomic DNA of *P*. *triticina* (pathotype 77–5) in PCR grade water. (B) Melting curve obtained through real time PCR products using *P*. *triticina* specific primers, no peak was observed in negative control.

The optimum annealing temperature for amplification using real time PCR was 51°C. The standard curve drawn using *P*. *triticina* DNA showed a linear correlation between Ct value and DNA concentration (fg), with a correlation coefficient of 0.997, showing the accuracy of real time PCR based quantification. Target DNA showed fluorescence which was not observed in negative controls. Amplification efficiency of the primers was 111.97% indicated by slope values of standard equations. First fluorescent signals were observed at Ct 33 corresponding approximately to 10 fg DNA, while at Ct 11.3 it reached to highest concentration (100 ng). Real time based marker showed the sensitivity of 100fg corresponding to Ct value 30.6 (Fig 4A). Specificity of the primers was confirmed by melting curve showing one distinct peak (Fig 4B).

### 3.3 Standardization of LAMP detection assay

The optimised concentration of different components in 25μl reaction buffer are: 2.5 μl of 10X thermo pol buffer, 8U of *Bst polymerase*, 4μl MgSO_4_ (25mM), 3 μl dNTPs (10mM), Betaine 5μl (Sigma), 20μM each of FIP, BIP, LF and LB primers, 5μM of each of F3 and B3 primers. Temperature gradient showed that reaction mix incubated at 65^0^ C for 60 mins was optimum for the assay developed in this study. Visual detection of LAMP reaction using 120μM HNB dye was optimum for colour change from violet to sky blue in positive reaction. At 0.6mg of EtBr /ml, brightness of the positive reaction tube was more as compared to the negative reaction visualised on transilluminator.

#### 3.3.1 Specificity and sensitivity test of LAMP assay

**Fig 5:**
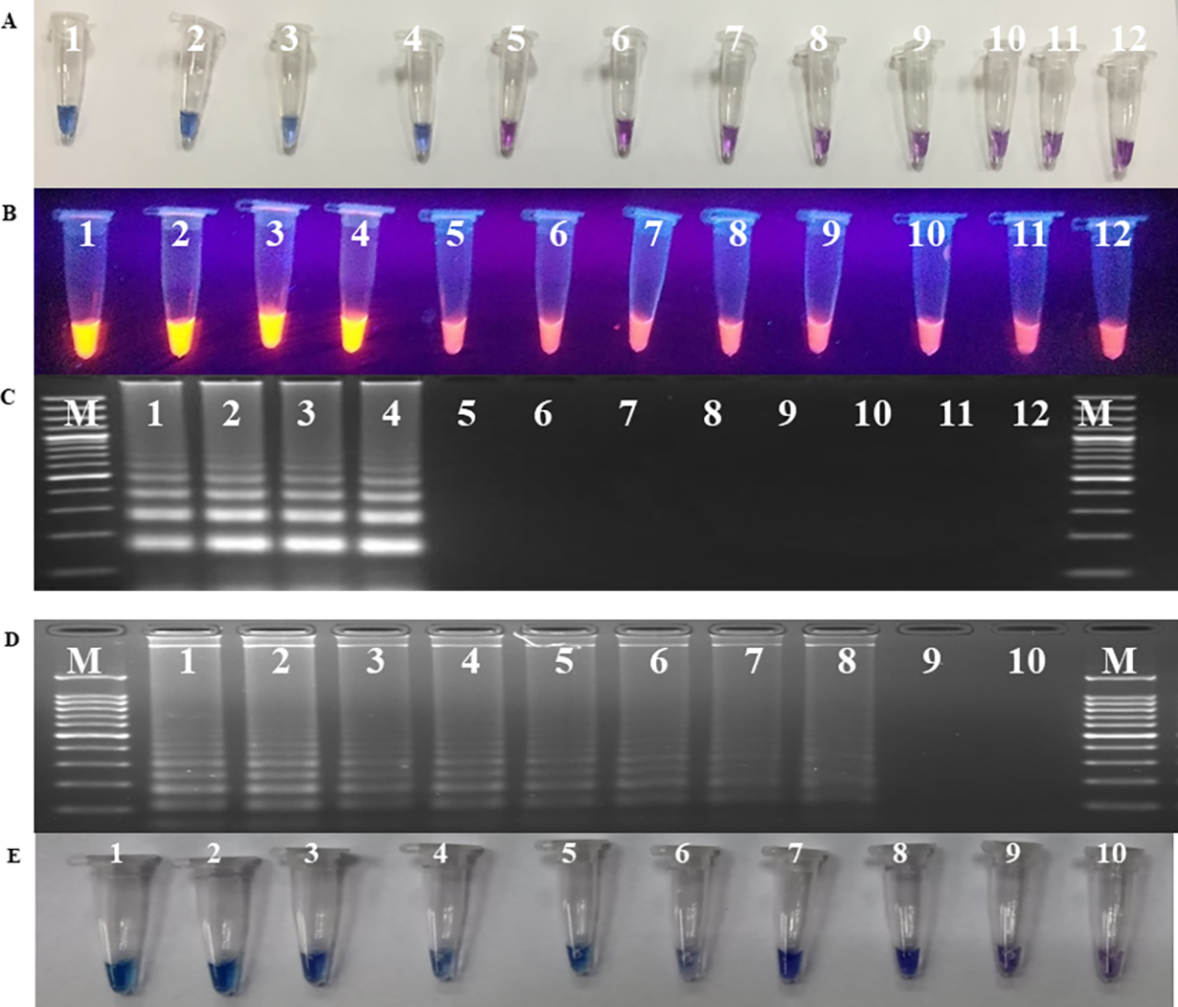
**Testing the specificity (A, B and C), and sensitivity (D and E) of LAMP assay.** A. Visual detection of LAMP reaction using HNB dye; B. Visual detection of LAMP reaction using EtBr dye; C. Agarose gel electrophoresis of LAMP products. Lanes DNA template of *Puccinia triticina* (1-12-2; 2–77–5; 3-104-2, 4-162-2); 5- *P*. *striiformis* f. sp. *tritici* (46S119); 6- *P*. *graminis* f. sp. *tritici* (40–1); 7-*Bipolaris sorokiniana*; 8- *B*. *oryzae*; 9- *Blumeria graminis tritici*; 10- *Fusarium graminearum*; 11-Wheat leaf DNA; 12- sterile water. (D) Sensitivity test using agarose gel electrophoresis; (E) Visual detection of LAMP reaction using HNB dye. Lanes: 1(100ng); 2 (50ng); 3 (10ng); 4 (1ng); 5 (100pg); 6 (50pg); 7 (10pg); 8 (100fg); 9 (10 fg); 10 (sterile water); M (100 bp molecular marker, Fermentas).

For testing specificity of LAMP, assay was performed with template DNA of *P*. *triticina*, *P*. *striiformis* f. sp. *tritici*, *P*. *graminis* f. sp. *tritici*, *B*. *sorokiniana*, *B*. *oryzae*, *Blumeria graminis tritici* and *F*. *graminaerum* as well as DNA isolated from healthy wheat sample. Under standardized conditions, amplification was found only in *P*. *triticina* and no amplification was observed in any other sample. Same results were witnessed by HNB dye based detection assay (Fig 5A), EtBr (Fig 5B) and gel electrophoresis (Fig 5C). The sensitivity of the LAMP detection assay was 100fg observed by DNA laddering pattern in agarose gel electrophoresis (Fig 5D). Similar results were also obtained by using HNB dye (Fig 5E).

#### 3.3.2 Validation and utilization of LAMP assay for diagnosis of leaf rust of wheat

**Fig 6:**
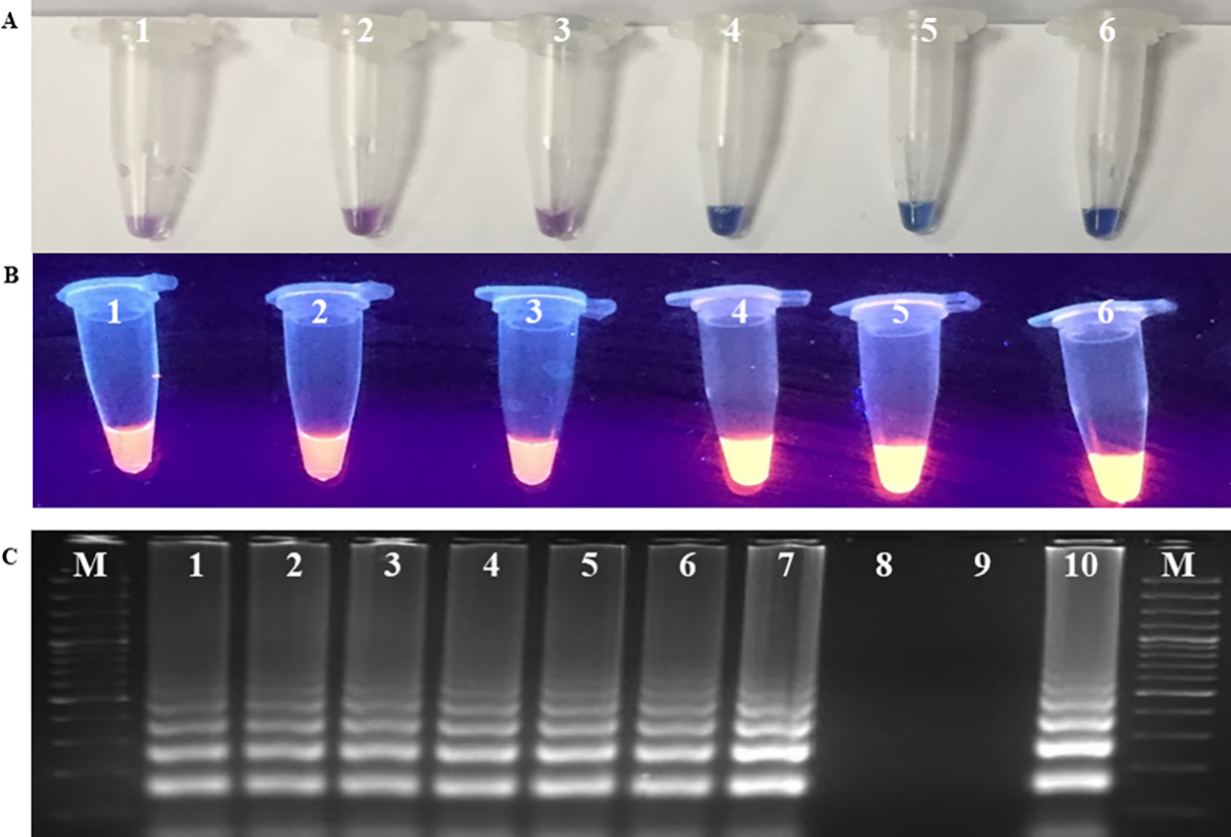
**Validation (A and B) and utilization of LAMP assay (C) for diagnosis of leaf rust of wheat.** A. Visual detection of LAMP reaction using HNB dye; B. Visual detection of LAMP reaction using EtBr dye; Lanes1- sterile water; 2-DNA from uninoculated healthy plant; 3- 0h 4- 24h, 5- 48h, 6- DNA template of *Puccinia triticina* (positive control). C. Utilization of LAMP assay for diagnosis of leaf rust in field samples using agarose gel electrophoresis. Lanes: M: 100bp molecular marker, Fermentas; 1–7: DNA template of wheat leaf rust infected samples; 8-healthy wheat leaf DNA; 9- sterile water; 10- DNA template of *Puccinia triticina* (positive control).

LAMP assay showed amplification of DNA in wheat leaves of susceptible variety (Agra local) collected at 24 hpi, 48 hpi along with positive control and no amplification was observed from DNA of uninoculated plant and negative control (NTC) (Figs 6A and 6B). All suspected samples taken randomly from field were found positive with amplification in LAMP assay developed in the study. This result was confirmed by gel electrophoresis (Fig 6C). LAMP based detection assay developed in study can be directly utilized for early and specific detection of *Puccinia triticina* in wheat.

## 4. Discussion

For effective management of leaf rust of wheat timely detection and forecasting of disease have paramount importance. Detection of *Puccinia triticina* by conventional methods is time consuming and labour intensive. Molecular detection methods are rapid, more sensitive and large number of samples can be tested at a time. Recently many plant pathogens have been diagnosed using PCR based detection assays, to mention few *P*. *striiformis* f. sp *striiformis* [2, 3], *P*. *graminis* f. sp. *tritici* [6], *B*.*s sorokianiana* [4], and *F*. *graminarium* complex [5]. Similarly in the present study we have developed PCR based detection assay for early and accurate diagnosis of leaf rust of wheat. The marker (PtRA_68_) developed can detect *P*. *triticina* in pre-symptomatic condition at 24 hpi hence we can take prophylactic fungicidal spray for better management of leaf rust of wheat.

For wheat breeder it is very much important to score the disease at different time intervals till its maturity during the screening of germplasm.Unfortunately we do observe simultaneous occurrence of all the three rust or two rusts in combination in the field and later they merge with each other. Under such conditions breeders cannot identify leaf rust of wheat specifically and cannot give accurate score for the germplasm for which they are breeding. Under this situation we need high through put DNA based detection protocols so that breeders can precisely identify and quantify leaf rust, so that right germplasm will be selected. To address this we have developed a PCR based marker (PtRA_68_F/ PtRA_68_R) which can specifically detect *P*. *triticina* by producing the band size 919 bp (Sequence Id: PtRA_68_: KY747393), which can differentiate *P*. *triticina* from other species of *Puccinia* and other wheat pathogens. Therefore, PCR based diagnostic assay developed in our study will definitely assist wheat breeders in selecting leaf rust resistant line during screening of germplasm and assist in development of leaf rust resistant wheat varieties.

Apart from development of marker we also validated it by collecting suspected leaf rust samples from research farms of ICAR-Indian Agricultural Research Institute, New Delhi and found three samples positive out of four. This data demonstrated that marker developed in the study is accurate and can be used for the samples collected directly from field.

The marker developed in study can detect the pathogen in an inoculum as low as 50 pg, therefore, it is more sensitive than our conventional methods. Further, we have developed qPCR based detection assay to quantify the inoculum load of pathogen and we found that it is much more sensitive (100 fg) than conventional PCR. Here an increase in sensitivity from the classical PCR method (50 pg) to qPCR (100 fg) was done by converting conventional PCR into qPCR. Similar results on detection of *Trichoderma atroviride* [21] and *Trichoderma harzianum* [22] using qPCR have been reported.

So by using the qPCR based detection assay we can quantify the primary inoculum load of leaf rust pathogen in the off season and can develop prediction models for early and accurate forecasting of leaf rust of wheat. In India, if we quantify the inoculum load in off season from Nilgiri and Palney hills which are considered to be inoculum loci for entire India [23] we can precisely say the possibility of leaf rust outbreak in next regular (*rabi*) season, so that farmers will have time to choose suitable resistant variety, can decide the time of sowing and other package of practices.

Although PCR based detection assays are most accepted and widely used for detection of plant pathogens, loop mediated isothermal amplification of DNA (LAMP) has many favourable advantages over PCR. LAMP assay amplifies DNA at a single temperature, hence there is no need of expensive equipment like thermal cycler but a simple dry bath is enough to perform a LAMP protocol. In the present study we optimized the LAMP based detection assay for *P*. *triticina* using dry bath. By this we simplified leaf rust diagnostic protocol without the requirement of sophisticated facility. Earlier, LAMP based detection assays have been developed for *P*. *striiformis* f. sp *tritici* [8], *Plasmopara viticola* causing downy mildew of grapes [24], *F*. *asiaticum* causing Fusarium head blight of wheat [25] and *Tilletia indica* causing Karnal bunt of wheat [26].

LAMP method can amplify DNA in 45 to 60 minutes which is much lesser than PCR, hence it is more rapid [9]. Since LAMP amplifies DNA in isothermal conditions and detect the pathogen visually, both amplification and detection can be completed in a single step. Change in the turbidity due to the formation of magnesium pyrophosphate during reaction in the amplified products (positive reaction) eliminates the requirement of running the amplified products in the gel electrophoresis for confirmation of results [11]. In the present report we observed the milky white color in the *Puccinia triticina* samples compared to other non-target pathogens where it was highly transparent (negative reaction), therefore LAMP based assay is visual method wherein amplification of DNA can be detected by naked eye. Major drawback of PCR is the chances of false positive since it uses only two primers and recognises only two regions of amplicon, whileLAMP utilizes six set of primers, it recognizes eight different regions of gene of interest, therefore, it is highly specific and by this we are eliminating the possibility of false positive reaction. In our study, LAMP specifically amplified only *P*. *triticina* pathotypes and no amplification was observed in other *Puccinia* species or other wheat pathogens. Therefore LAMP based detection assay is more specific and reliable method for early and accurate diagnosis of leaf rust of wheat.

In agreement with Ghosh et al 2017 [27], we also observed that LAMP is more sensitive than conventional PCR. In the present study LAMP assay detected *P*. *triticina* at 100 fg concentration of DNA although it was same as qPCR but conventional PCR could detect pathogen DNA at 50 pg.To demonstrate the application of LAMP for field based diagnosis, we collected the suspected leaf rust samples from ICAR-Indian Agricultural Research Institute, New Delhi research farm and observed that all the infected samples along with positive control were amplified. Therefore, LAMP based detection assay is a field based assay and it can be utilized for screening germplasm directly from field.

Further loop mediated isothermal amplification method of detection of *P*. *triticina* was made colorimetric assay by supplementing reaction with HNB and EtBr. HNB is a colorimetric indicator of calcium and alkaline earth metal ions. In comparison with other methods of visual detection, the use of HNB is simpler and useful [28]. In the present study we observed change in color from violet to sky blue in positive reaction when we add HNB to reaction mixture, similar results were reported in chickpea wilt [8, 29].

Addition of EtBr to the reaction mixture showed the difference in the brightness between positive and negative reaction on UV transilluminator. Reaction mixture having genomic DNA of *P*. *triticina* showed full brightness in comparison with other *Puccinia* spp and non-target pathogens, Similar results were obtained in LAMP based detection of stripe rust of wheat [8]. Hence, LAMP based detection assay is a colorimetric assay, less time consuming and requires no gel electrophoresis.

Since LAMP assay is simple, colorimetric, rapid, more sensitive it suits better for development of diagnostic kit for early and accurate detection of *P*. *triticina*, forecasting of leaf rust of wheat based on inoculum load in off season, development of prediction models for outbreak of disease and better management of the disease by taking prophylactic fungicidal spray.

## 5. Conclusion

Here we report, for the first time specific molecular assay for early detection of leaf rust of wheat pathogen, *Puccinia triticina* through conventional PCR, qPCR as well as loop mediated isothermal amplification of DNA (LAMP). The developed assay helps in early and rapid detection of the pathogen on wheat leaves with high sensitivity. LAMP assay developed shall also help in monitoring of the pathogen which will contribute to the accurate forecast and precise control of this disease.

### Availability of data

The nucleotide sequence data (*Puccinia triticina* specific marker: PtRA_68_) reported in the manuscript is available in the NCBI database (https://www.ncbi.nlm.nih.gov) under the accession no. KY747393.

## References

[1] Bolton MD, Kolmer JA, Garvin DF. Wheat leaf rust caused by *Puccinia triticina*. Molecular plant Pathology. 2008; 9(5): 563–575. doi: 10.1111/j.1364-3703.2008.00487.x 19018988 10.1111/j.1364-3703.2008.00487.x PMC6640346

[2] Aggarwal R, Sharma S, Gupta G, Manjunatha C., Singh V K., Kulshreshtha D. Gene based analysis of Puccinia species and development of PCR based marker for the detection of *Puccinia striiformis* f. sp. *tritici* causing yellow rust of wheat. Journal of General Plant Pathology. 2017; 83:205–215.

[3] Lihua C, Shichang X, Ruiming L, Taiguo L, Wanquan C. Early molecular diagnosis and detection of *Puccinia striiformis* f. sp. *tritici* in China. Letters in Applied Microbiology. 2008; 46:501–506. doi: 10.1111/j.1472-765X.2007.02313.x 18363658 10.1111/j.1472-765X.2007.02313.x

[4] Aggarwal R, Gupta S, Banerjee S, Singh VB. Development of a SCAR marker for detection of *Bipolaris sorokiniana* causing spot blotch of wheat. Canadian Journal of Microbiology. 2011; 57: 934–942. doi: 10.1139/w11-089 22017748 10.1139/w11-089

[5] Hamada MS, Yin YN, Ma ZH. Rapid detection of *Fusarium graminearum* complex in wheat seeds using species-specific PCR primer designed based on a microsatellite region. Cereal Research Communications. 2012; 40: 85–94.

[6] Liu TG, Wang X, Gao L, Liu B, Chen WQ, Xiang WS. A FIASCO based approach for detection and diagnosis of *Puccinia graminis* f. sp. *tritici* in China. Journal of Integrative Agriculture. 2014; 13: 2438–2444.

[7] Kudo YH, Yoshino M, Kojima T, Ikedo M. Loop-mediated isothermal amplification for the rapid detection of Salmonella. FEMS Microbiology Letters. 253 (1): 155–161. doi: 10.1016/j.femsle.2005.09.032 16242860 10.1016/j.femsle.2005.09.032

[8] Aggarwal R, Sharma S, Manjunatha C, Gupta S, Singh VK. Development and validation of loop mediated isothermal amplification based detection assay for *Puccinia striiformis* f. sp. *tritici* causing stripe rust of wheat. Australasian Plant Pathology. 2017; 46: 577–583.

[9] Notomi T, Okayama H, Masubuchi H, Yonekawa T, Watanebe K, Amino N, et al Loop mediated isothermal amplification of DNA. Nucleic Acids Research. 2000; 28: E63 10871386 10.1093/nar/28.12.e63 PMC102748

[10] Kuan C, Wu M, Lu Y, Huang H. Rapid detection of squash leaf curl virus by loop-mediated isothermal amplification. Journal of Virology Method. 2010; 169: 61–65. 10.1016/j.jviromet.2010.06.017 20603151

[11] Fukuta S, Mizukami Y, Ishida A, Ueda J, Hesegawa M, Hayashi I, et al Real time loop mediated isothermal amplification for the CaMV-35S promoter as a screening method for genetically modified organisms. European Food Research Technology. 2004; 218: 496–500.

[12] Das A, Babiuk S, McIntosh MT. Development of a Loop-Mediated Isothermal Amplification Assay for Rapid Detection of Capripoxviruses. Journal of Clinical Microbiology. 2012; 50(5):1613–1620. doi: 10.1128/JCM.06796-11 22357504 10.1128/JCM.06796-11 PMC3347125

[13] Xie L, Xie Z, Zhao G. A loop-mediated isothermal amplification assay for the visual detection of duck circovirus. Virology Journal. 2014; 11:76 doi: 10.1186/1743-422X-11-76 24775810 10.1186/1743-422X-11-76 PMC4013541

[14] Parida M, Sannarangaiah S, Dash PK. Loop mediated isothermal amplification (LAMP): a new generation of innovative gene amplification technique; perspective in clinical diagnosis of infectious diseases. Reviews in medical virology. 2008; 18(6): 407–421. doi: 10.1002/rmv.593 18716992 10.1002/rmv.593 PMC7169140

[15] Buhlmann A, Pothier J, Rezzonico F, Smits THM, Andreou M, Boonham N, et al *Erwinia amylovora* Loop mediated isothermal amplification (LAMP) assay for rapid pathogen detection and on site diagnosis of fire blight. Journal of Microbiology Method. 2013;92: 332–339. 10.1016/j.mimet.2012.12.017 23275135

[16] Kubota R, Vine BG, Alvarez AM, Jenkins DM. Detection of *Ralstonia solanacearum* by Loop-mediated isothermal amplification. Phytopathology. 2008; 98: 1045–1051. doi: 10.1094/PHYTO-98-9-1045 18943743 10.1094/PHYTO-98-9-1045

[17] Obura E, Masiga D, Wachira F, Gurja B, Khan ZR. Detection of phytoplasma by loopmediated isothermal amplification of DNA (LAMP). Journal of Microbiological Method. 2011; 84: 312–316. 10.1016/j.mimet.2010.12.011 21185882

[18] Nie X. Reverse transcription loop-mediated isothermal amplification of DNA for detection of Potato virus Y. Plant Disease. 2005; 89: 605–610. 10.1094/PD-89-0605 30795385

[19] Murray MG, Thompson WF. Rapid isolation of high molecular weight plant DNA. Nucleic Acids Research. 1980; 8: 4321–4326. 7433111 10.1093/nar/8.19.4321 PMC324241

[20] Szabo- Les J, Kolmer JA. Development of simple sequence repeat markers for the plant pathogenic rust fungus *Puccinia triticina*. Molecular Ecology Notes. 2007; 7: 708–710.

[21] Savazzini F, Longa CMO, Pertot I, Gessler C. Real-time PCR for detection and quantification of the biocontrol agent *Trichoderma atroviride* strain SC1 in soil. Journal of Microbiology Methods. 2008; 73: 185–194. 10.1016/j.mimet.2008.02.004 18375004

[22] Sanzani SM, Li Destri Nicosia MG, Faedda R, Cacciola SO, Schena L. Use of quantitative PCR detection methods to study biocontrol agents and phytopathogenic fungi and oomycetes in environmental samples. Journal of Phytopathology. 2014; 162: 1–13.

[23] Nagarajan S, Joshi LM. Further Investigations on Predicting Wheat Rusts Appearance in Central and Peninsular India. Phytopathology. 1980; Z 98: 84–90.

[24] Kong XJ, Qin WT, Huang XQ, Kong FF, Schoen CD, Feng J. Development and application of loop-mediated isothermal amplification (LAMP) for detection of *Plasmopara viticola*. Scientific Reports. 2016; 6: 28935 doi: 10.1038/srep28935 27363943 10.1038/srep28935 PMC4929445

[25] Xu M, Ye W, Zeng D. Rapid diagnosis of wheat head blight caused by *Fusarium asiaticum* using a loop-mediated isothermal amplification assay Australasian Plant Pathology. 2017.

[26] Gao Y, Tan MK, Zhu YG. Rapid and specific detection of *Tilletia indica* using loop-mediated isothermal DNA amplification. Australasian Plant Pathology. 2016; 45:361–367.

[27] Ghosh R, Tarafdar A, Mamta S. Rapid and sensitive diagnoses of dry root rot pathogen of chickpea (Rhizoctoniabataticola (Taub.) Butler) using loop-mediated isothermal amplification assay. Scientific Reports. 2017; 7:42737 doi: 10.1038/srep42737 28218268 10.1038/srep42737 PMC5316965

[28] Goto M, Honda E, Ogura A, Nomoto A, Hanaki K. Colorimetric detection of loop-mediated isothermal amplification reaction by using hydroxy naphthol blue. BioTechniques. 2009; 46:167–172. doi: 10.2144/000113072 19317660 10.2144/000113072

[29] Ghosh R, Nagavardhini A, Sengupta A, Sharma M. Development of Loop-Mediated Isothermal Amplification (LAMP) assay for rapid detection of *Fusarium oxysporum* f. sp. *ciceris*—wilt pathogen of chickpea. BMC Research Notes. 2015; 8:40 doi: 10.1186/s13104-015-0997-z 25886622 10.1186/s13104-015-0997-z PMC4332723

